# A method of precise mRNA/DNA homology-based gene structure prediction

**DOI:** 10.1186/1471-2105-6-261

**Published:** 2005-10-21

**Authors:** Alexander Churbanov, Mark Pauley, Daniel Quest, Hesham Ali

**Affiliations:** 1 Department of Computer Science, College of Information Science and Technology, University of Nebraska at Omaha, Omaha, NE 68182-0116, USA

## Abstract

**Background:**

Accurate and automatic gene finding and structural prediction is a common problem in bioinformatics, and applications need to be capable of handling non-canonical splice sites, micro-exons and partial gene structure predictions that span across several genomic clones.

**Results:**

We present a mRNA/DNA homology based gene structure prediction tool, GIGOgene. We use a new affine gap penalty splice-enhanced global alignment algorithm running in linear memory for a high quality annotation of splice sites. Our tool includes a novel algorithm to assemble partial gene structure predictions using interval graphs. GIGOgene exhibited a sensitivity of 99.08% and a specificity of 99.98% on the Genie learning set, and demonstrated a higher quality of gene structural prediction when compared to Sim4, est2genome, Spidey, Galahad and BLAT, including when genes contained micro-exons and non-canonical splice sites. GIGOgene showed an acceptable loss of prediction quality when confronted with a noisy Genie learning set simulating ESTs.

**Conclusion:**

GIGOgene shows a higher quality of gene structure prediction for mRNA/DNA spliced alignment when compared to other available tools.

## Background

A vast amount of genomic data, including most of the human genome [1], is now available in publicly accessible databases, and the deposition of additional data continues at a rapid pace. Genomic data requires meticulous interpretation and annotation for meaningful information to be extracted. Genes, the most important functional blocks in the human genome, require exact structural annotation for future biological experiments such as reverse genetics and microarray experiments.

Most of the human genes have piecewise structure with a number of exons separated by introns. Introns are excised from original gene transcripts (pre-mRNA) to form mature mRNA. By aligning mRNA with originating genomic clones, we can reconstruct gene structure.

Several fast and efficient tools, such as BLAST [2] and BLASTX [3], were introduced in the early 90's to search databases for homologous blocks, an essential component of all gene structural prediction algorithms. An original method of gene structure prediction based on a set of protein-DNA blocks [4], implemented in GeneBuilder, was followed by Procrustes implementation [5]. Later, there were numerous implementations exploiting the idea of homology-based gene structure prediction, including GeneSeqer with SplicePredictor [6], AAT [7], EbEST [8], ESTMAP [9], TAP [10], Sim4 [11], Spidey [12], GrailEXP Galahad [13], BLAT [14] and est2genome [15]. Other genome annotation software is described in [16,17].

Homology-based methods of gene structure prediction, referred to as *spliced alignment*, are often classified according to the homology type they employ (DNA/DNA, DNA/mRNA, DNA/Protein, etc.) [16]; frequently, programs employ more than one homology type. The purpose of a spliced alignment algorithm is to explore all possible assemblies of potential exons (blocks) to find a chain of exons which best fits an mRNA target sequence.

In this paper we discuss GIGOgene, a gene structure prediction tool. GIGOgene, like existing spliced alignment software [11,16], can deal with repeating domains, paralogs and pseudogenes. In addition, GIGOgene is capable of combining structural prediction of a gene from partial gene models that span across several genomic clones. The key to GIGOgene higher precision, in the case of mRNA/DNA spliced alignment, is in the use of new splice-enhanced affine gap penalty global alignment for noise-tolerant recovery of exon-intron boundaries, including non-canonical splice sites, with simultaneous prediction of short exons. GIGOgene uses a filtering step to remove suboptimal blocks for better prediction quality.

## Implementation

Before we proceed with formal description of methods, we need to define a *High-scoring Segment Pair *(HSP), otherwise known as a *block*. In the context of this paper, an HSP is a statistically significant alignment between *segments *(subsequences) in DNA and mRNA obtained from a BLASTN result. Parameters characterizing HSPs include location in the mRNA query and in the DNA target sequence, and different quality values such as expectation value (E), percent identity, and score.

Below, we provide a brief description of the steps in our gene structural prediction process. Some of these steps are self-explanatory, while others are considered in detail in the following subsections:

**Step 1 **Align curated mRNA sequence(s) with DNA target sequence database using BLASTN [2].

**Step 2 **Parse the BLASTN output and select genomic clones that score above 200 bits with an expectation value of no more than 1*e*1. Through experimentation we have determined that these values are optimal for recovery of most of the essential HSP sets needed for further analysis. These values can be easily adjusted.

**Step 3 **By pairwise comparative analysis of an HSP set for each selected genomic clone, exclude HSPs with mRNA segments totally within other larger mRNA HSP segments. The longest HSP is assumed to contain the true exonic boundaries; shorter subHSPs usually result from paralogous and pseudogenic matches.

**Step 4 **Disambiguate the HSP sequences for all the selected clones, as discussed [see Subsection *Algorithm for an unambiguous HSP sequences allocation*]. The result of this step is a set of unambiguous HSP sequences.

**Step 5 **Build an *interval graph *of overlapping unambiguous HSP sequences. The interval graph captures intersection relations of nodes (unambiguous HSP sequences) as we put edges between nodes when nodes belong to different genomic clones, while their mRNA composite segments intersect. Edges between HSP sequences from the same genomic clone are not allowed.

**Figure 1:**
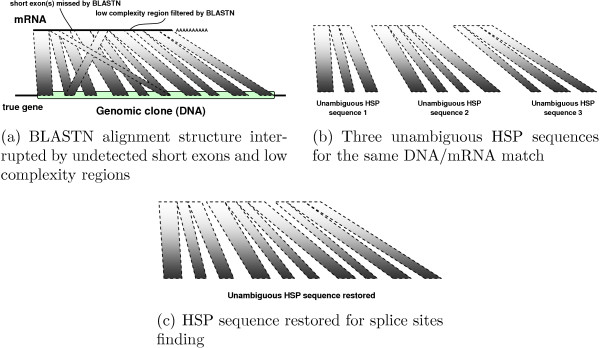
Schematic example of HSP sequence restoration.

**Step 6 **Occasionally, short exons missed by the BLASTN algorithm or dust low-complexity filtering result in interrupted unambiguous HSP sequences. Their fragments reside in different interval graph nodes marked with the same genomic clone and transcript. We merge these nodes to form longer, original, uninterrupted unambiguous HSP sequences. An intuitive interpretation of this step is in Figure 1.

**Step 7 **Compact the interval graph, as discussed [see Subsection *Joining unambiguous HSP sequences*]. This results in the biggest composite genomic clone containing the maximum number of possible exons.

**Step 8 **Use splice-enhanced affine gap penalty global alignment to identify possible intron/exon boundaries in the composite genomic clone, as discussed [see Subsection *Splice-enhanced affine gap penalty global alignment*].

**Step 9 **Extract intron and exon segments from the composite genomic clone and print a report.

### Algorithm for an unambiguous HSP sequences allocation

**Figure 2:**
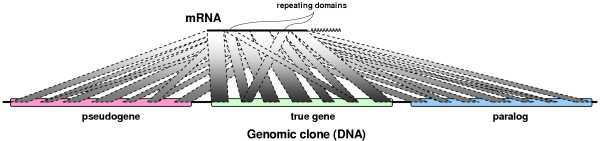
BLASTN alignment structure interpretation. Matches to pseudogene(s) and paralog(s) may be misinterpreted as resulting from original gene; repeating domains in mRNA further confuse gene structure prediction by causing cross-matches.

It is well-known that genes, or parts of genes, are duplicated during the course of evolution. This can result in ambiguities during the assembly of a complete gene structure from HSP sequences, as illustrated in Figure 2.

The problem arises when a segment in an mRNA transcript matches multiple segments in a genomic clone. To address this our algorithm for finding an unambiguous HSP sequence (a chain of putative exons) adheres to the following biological principles:

1. Transcripts are always linear. Thus, we require the set of HSPs to be sequential (we refer to this as the *sequential rule*).

2. Splicing of pre-mRNA does not introduce any alternations in the order of exons.

3. Alternative splicing does not affect the order of exons in a gene.

4. The similarity of homologous fragments of a gene gradually decreases due to sporadic mutations. As a result, HSPs from the real gene usually have higher scores than HSPs from corresponding pseudogene(s) or paralog(s), as schematically shown in Figure 2. We thus reject unambiguous HSP sequences with average percent identities below a certain threshold; a threshold of 97% produced good results in our experiments with mRNAs. Threshold value could be easily adjusted, if needed, to find gene structure with distant homologs, such as Expressed Sequence Tags (ESTs).

5. Pre-mRNA splicing results in mature mRNA, with exons arranged side by side. In the case of an HSP sequence containing potential exons, we require the entire mRNA transcript to be covered with segments continuously, without breaks.

**Figure 3:**
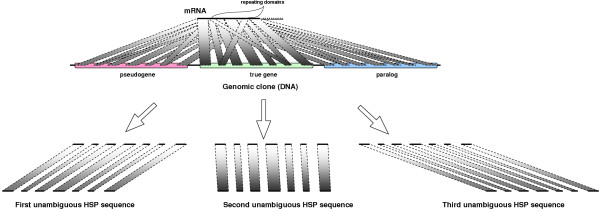
Idea behind the disambiguating algorithm. We distinguish HSP sequences matching real gene, pseudogene(s) and paralog(s), eliminating HSPs from repeating domains.

Disambiguation of an HSP set is shown in Figure 3.

**Figure 4:**
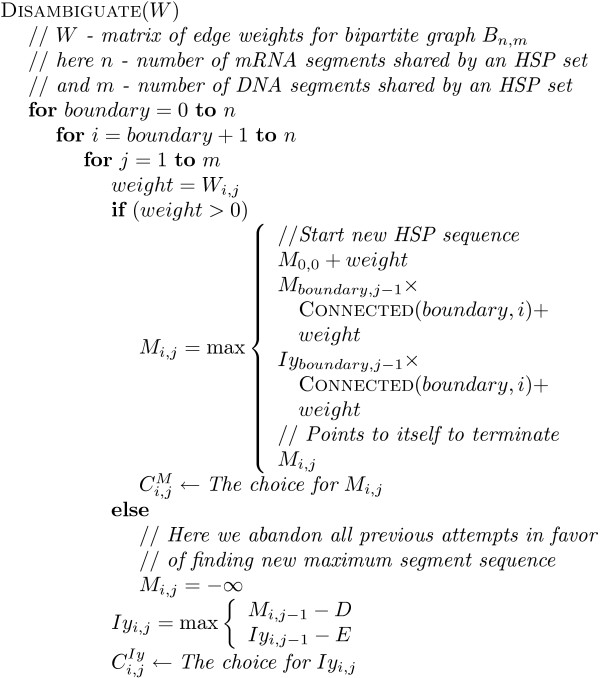
HSP sequence disambiguating algorithm.

For the purposes of the disambiguating algorithm we build a bipartite graph structure, where segments are nodes and HSPs are edges connecting the nodes. A dynamic programming disambiguation procedure with an affine gap penalty (Figure 4) is then used to disambiguate the HSPs into a linear sequence. Modifying our early system prototype [18], we changed the criteria for solution optimality (we originally estimated solution quality based on average HSP sequence identity).

In our experiments, we noticed cases in which HSPs from the real gene match have a smaller identity compared to HSPs originating from paralogs and pseudogenes. Thus, the disambiguation procedure finds the unambiguous HSP sequence covering the longest mRNA segment with the minimum number of HSPs. For the HSP sequences of equal length with the same number of HSPs, we compare the maximum total *weight *where the weight of an HSP is a tradeoff between its identity and size:

*weight = size·m*^100-*x *^    (1)

Here *x *is the BLASTN-assigned percent identity for an HSP, *size *is the HSP length, and *m <*1 is the *decay rate *to ensure substantial weight loss for identity lower than the threshold value. The value *m = *0.85 produced good results in our experiments. Weight function (1) characterizes the importance of any given HSP in a global solution.

**Figure 5:**
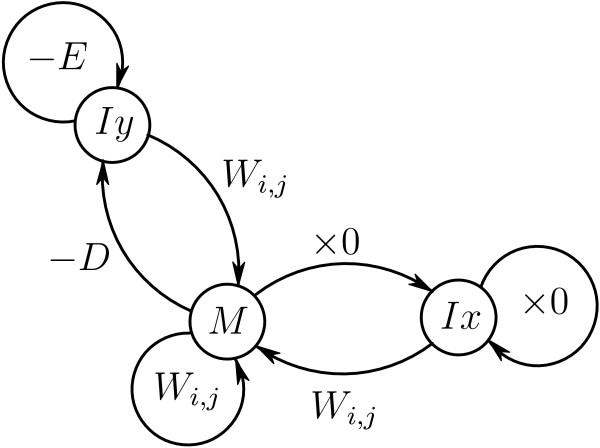
State Diagram of the disambiguating algorithm. Here *W*_*i*,*j *_is the weight (1) of an HSP containing transcript segment *i *and genomic clone segment *j*. States correspond to weight matrices of partial solutions in dynamic programming.

The disambiguation procedure can be represented as a series of transitions between states (Figure 5). State *Iy *is visited when a sequence of genomic segments is interrupted. This subtracts *D*, and *E *for every additional genomic segment missed, from the total weight. If a continuously overlapping transcript-side sequence of segments is broken, the total weight is nullified by visiting state *Ix*. Weight is gained at state *M *with a normal transcript-side overlapping sequence of HSPs.

For this algorithm we must allocate a score matrix *F *of dimensionality 2 × (*# of RNA segments*) × *(# of DNA segments) *and a matrix *C *of the same size to record the intermediate HSP sequences in the dynamic programming procedure. For the convenience of indexing we introduce aliases M ← *F*_0 _and *Iy *← *F*_1_. We ignore matrix *Ix *as being unnecessary. The boolean function CONNECTED(*i*, *j*) determines the overlap between segments *i *and *j *in the transcript.

**Figure 6:**
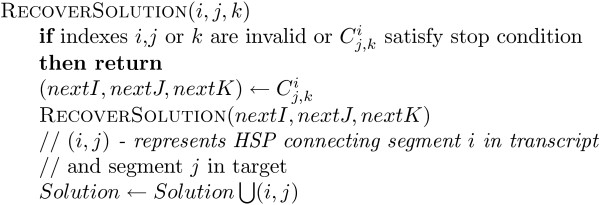
Solution recovery.

To generate the final set of unambiguous HSP sequences for a given BLASTN result, the HSP sequences are restored from matrix *C *using the recursive algorithm shown in Figure 6. At the end of the disambiguating procedure we disregard HSP sequences of average identity lower than threshold value. As explained below, the resulting set of unambiguous HSP sequences can then be optimally connected using an interval graph.

### Joining unambiguous HSP sequences

**Figure 7:**
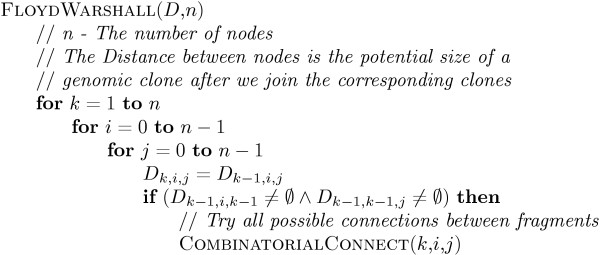
Modified Pairwise Floyd-Warshall.

To construct a complete HSP sequence out of several smaller overlapping ones, we build an interval graph. Each node in the graph is an unambiguous HSP sequence originating from the disambiguating algorithm discussed [see Subsection *Algorithm for an unambiguous HSP sequences allocation*]. In order for the nodes to be connected by an edge, they must contain overlapping HSP sequences coming from different genomic clones. To join the nodes, a Floyd-Warshall all-pairs-longest-path algorithm [19] known to run in *O*(*n*^3^) time is used (Figure 7). Joining nodes provides both a larger HSP sequence and the ability to join two genomic clones at a common point.

**Figure 8:**
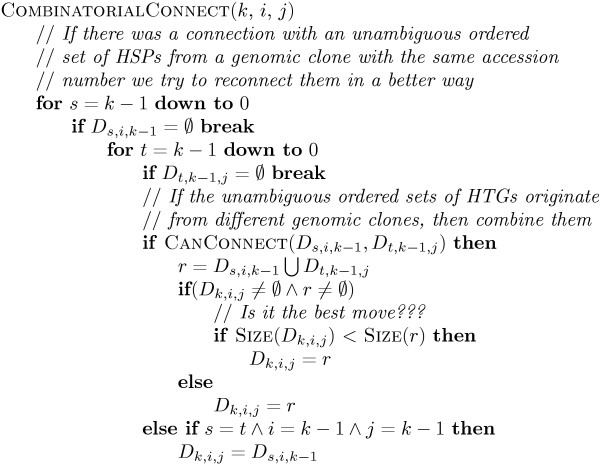
Finding the best combination of HSP sequences to connect.

If an attempt is made to connect nodes with overlapping HSP sequences from the same genomic clone, the program backs up and searches for other possible optimal unambiguous connections for different clones (see the algorithm in Figure 8). This backing-up modification adds at most *O*(*n*^2^) for each step in the pairwise algorithm for a dense graph, resulting in an *O*(*n*^5^) procedure.

Although the produced graphs may have different degrees of density, in our experiments they were not sparse enough to use Johnson's modification [19], which runs in *O*(*V*^2 ^log *V *+ *VE*).

To connect the nodes, we solve the following maximization problem:

Dk,i,j={Combination HSP sequences i and j, if k=0,argmaxDk−1,i,j,Dk−1,i,k∪Dk−1,k,j(max(SIZE(Dk−1,i,j),SIZE(Dk−1,i,k∪Dk−1,k,j))),if k>0.
 MathType@MTEF@5@5@+=feaafiart1ev1aaatCvAUfKttLearuWrP9MDH5MBPbIqV92AaeXatLxBI9gBaebbnrfifHhDYfgasaacH8akY=wiFfYdH8Gipec8Eeeu0xXdbba9frFj0=OqFfea0dXdd9vqai=hGuQ8kuc9pgc9s8qqaq=dirpe0xb9q8qiLsFr0=vr0=vr0dc8meaabaqaciaacaGaaeqabaqabeGadaaakeaacqWGebardaWgaaWcbaGaem4AaSMaeiilaWIaemyAaKMaeiilaWIaemOAaOgabeaakiabg2da9maaceaabaqbaeaabiGaaaqaaiabboeadjabb+gaVjabb2gaTjabbkgaIjabbMgaPjabb6gaUjabbggaHjabbsha0jabbMgaPjabb+gaVjabb6gaUjabbccaGiabbIeaijabbofatjabbcfaqjabbccaGiabbohaZjabbwgaLjabbghaXjabbwha1jabbwgaLjabb6gaUjabbogaJjabbwgaLjabbohaZjabbccaGiabdMgaPjabbccaGiabbggaHjabb6gaUjabbsgaKjabbccaGiabdQgaQbqaaiabbYcaSiabbccaGiabbMgaPjabbAgaMjabbccaGiabdUgaRjabg2da9iabicdaWiabcYcaSaqaamaawafabeWceaqabOqaaiabdseaenaaBaaabaGaem4AaSMaeyOeI0IaeGymaeJaeiilaWIaemyAaKMaeiilaWIaemOAaOgabeaacqGGSaalaeaacqWGebardaWgaaqaaiabdUgaRjabgkHiTiabigdaXiabcYcaSiabdMgaPjabcYcaSiabdUgaRbqabaGaeSOkIufabaGaemiraq0aaSbaaeaacqWGRbWAcqGHsislcqaIXaqmcqGGSaalcqWGRbWAcqGGSaalcqWGQbGAaeqaaaaaleqaneaacqqGHbqycqqGYbGCcqqGNbWzcqqGTbqBcqqGHbqycqqG4baEaaGcdaqadaqaaiabb2gaTjabbggaHjabbIha4naabmaabaqbaeqabiqaaaqaaiabbofatjabbMeajjabbQfaAjabbweafnaabmaabaGaemiraq0aaSbaaSqaaiabdUgaRjabgkHiTiabigdaXiabcYcaSiabdMgaPjabcYcaSiabdQgaQbqabaaakiaawIcacaGLPaaacqGGSaalaeaacqqGtbWucqqGjbqscqqGAbGwcqqGfbqrdaqadaqaauaabeqaceaaaeaacqWGebardaWgaaWcbaGaem4AaSMaeyOeI0IaeGymaeJaeiilaWIaemyAaKMaeiilaWIaem4AaSgabeaakiablQIivbqaaiabdseaenaaBaaaleaacqWGRbWAcqGHsislcqaIXaqmcqGGSaalcqWGRbWAcqGGSaalcqWGQbGAaeqaaaaaaOGaayjkaiaawMcaaaaaaiaawIcacaGLPaaaaiaawIcacaGLPaaaaeaacqGGSaalcqqGPbqAcqqGMbGzcqqGGaaicqWGRbWAcqGH+aGpcqaIWaamcqGGUaGlaaaacaGL7baaaaa@C70A@

Figure 7 shows the dynamic programming procedure, after we initialize matrix *D*. Upon completion of the procedure, we extract the maximum element from matrix *D*_*n *_and recover the solution. The COMBINATORIALCONNECT function used to find the best combination of HSP sequences is shown in Figure 8.

### Splice-enhanced affine gap penalty global alignment

In order to identify precise intron/exon boundaries in a genomic clone, a modified Needleman-Wunch global alignment algorithm with affine gap penalty is used to create a *spliced alignment *between segments of query and target sequences.

The basic Needleman-Wunch algorithm provides a scattered (i.e. frequently interrupted) mRNA/DNA alignment pattern, with no clear indication of exon/intron boundaries. With affine gap penalties, we penalize the score each time we break an alignment [20]; this provides an alignment clustered within putative exons, but usually without precise indications of exon/intron boundaries. The addition of sensor information (GT/AG, AT/AC or similar rules [21]) results in precise gene structural prediction.

**Figure 9:**
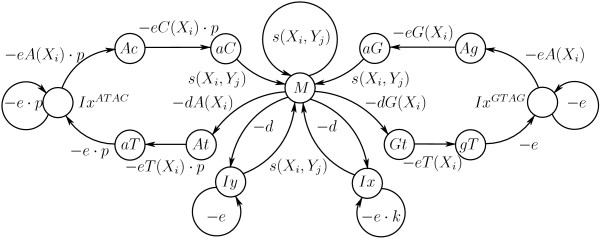
State Diagram of the modified spliced alignment algorithm. See the text for an explanation of the various states.

Our implementation is a modification of the affine gap penalty algorithm [20] and can be explained in terms of transitions between states in a Hidden Markov Model (HMM) [20]. Specifically, there are thirteen matrices of size *n *× *m *introduced, corresponding to states as shown in Figure 9. The matrices are reduced to arrays of size 2 × 13 × *m*, since we need only two rows in the scoring matrix *F *and backtracking matrices [22,23].

In our algorithm, we introduce the match state *M *(Figure 9), which uses the BLASTN scoring matrix. The state *Iy *corresponds to a gap penalty in the mRNA transcript, while the other states correspond to forming gaps in the genomic clone and have nucleotide-specific score deductions. Gap-opening matrices *dA *and *dG *express score preferences to open a gap with either nucleotide *A *or *G*, respectively; *d *is a generic gap-opening penalty; and *e *is a generic gap-extending penalty. Typically, the cost of extending a gap is set to be five to ten times lower than the cost for opening a gap. Gap-extension penalty matrices *eA, eC, eG *and *eT *express scoring preference to extend gaps with nucleotides *A*, *C*, *G *and *T*, respectively.

In order to save running time, we use *anchors *– short nucleotide segments from mRNA and DNA expected to contain exon/intron boundary fragments with donor/acceptor signals. A normal anchor does not have mismatches in state *M*. If a mismatch is encountered, it may mean a short exon is present. If necessary, the anchor can be expanded and the spliced alignment rerun with two full exons and intron between to identify possible short exons or address sequencing errors, as discussed [see Subsection *Advantages of using splice-enhanced affine gap penalty global alignment in gene structure prediction*].

According to our model, introducing or extending a gap is straightforward using the GT/AG rule. The penalty becomes severe if we try inserting a gap without the rule; we would rather use higher-extension-penalty state *Ix *for short gaps frequently resulting from sequencing errors. The AT/AC rule works in much the same way, except with a higher score penalty.

We implement the affine gap penalty spliced alignment algorithm in a linear memory of size *S*(*m *+ *n*) and running time *O*(*n *× *m*), where *n *is the size of a DNA fragment and *m *is the size of an mRNA fragment.

These are the steps in implementing the algorithm:

1. Run the spliced alignment ALIGN(0...*n*, 0...*m*) to find indexes of *u *and *v *(the split points for a recursive call). Here *u *is the vertical median index, and *v *is the horizontal index of a point where the optimal traceback intersects the median.

2. Restore the matrix context for the recursive calls and prior-state information for proper backtracking.

3. Make the recursive calls for nucleotide segments ALIGN(0...*u*, 0...*v*) and ALIGN(*u*...*n, v...m*), etc.

4. If either of the nucleotide segments' lengths in a recursive call is less than or equal to 1, call the ordinary spliced alignment for these pieces to get the alignment states.

More detailed explanation of the spliced alignment algorithm we use is in [18].

### Advantages of using splice-enhanced affine gap penalty global alignment in gene structure prediction

There are several advantages of using splice-enhanced affine gap penalty global alignment, discussed [see Subsection *Splice-enhanced affine gap penalty global alignment*], for gene structure prediction:

• ability to recover canonical and non-canonical splice sites;

• noise-tolerant prediction of splice sites;

• ability to recover short exons;

• ability to handle low complexity regions in genomic DNA, if sorted out by dust filtering.

A splice site usually happens on the boundaries of HSPs, but in most cases mRNA segments of neighboring HSPs overlap with no clear indication of a splice site. Recovery of a splice site could be formulated as a combinatorial problem of finding optimal exon/intron boundaries in HSPs' overlap vicinity. A dynamic programming approach, such as the splice-enhanced affine gap penalty global alignment we use, allows us to consider all possible rearrangements around HSPs' overlap to pick optimal splice sites in polynomial time.

**Figure 10:**
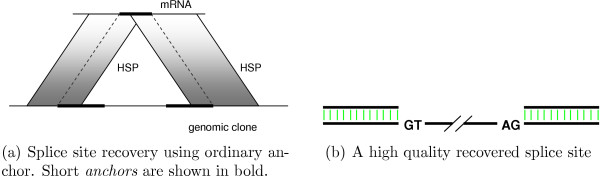
Recovery of optimal splice site.

The process of splice site recovery is schematically shown in Figure 10. Segments of mRNA and DNA sequences used for splice site prediction are called *anchors*. To accelerate the gene structure prediction process we use small (30 nt) anchors by default.

**Figure 11:**
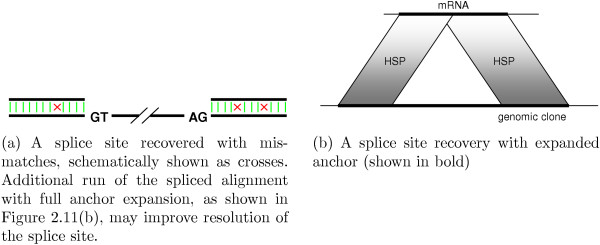
Recovery of suboptimal splice site.

The ideal variant of splice site recovery is shown in Figure 10. In a small number of cases we have misalignment, as shown schematically in Figure 11. Misalignment may occur if:

• neighboring HSPs overlap too much, so that we can't reliably identify a splice site with small anchors;

• there is a sequencing error adjacent to a splice site;

• short exons are present.

All of these cases require additional application of splice-enhanced affine gap penalty global alignment with anchors expanded to include the entire HSP segments for maximum error tolerance, as shown in Figure 11.

**Figure 12:**
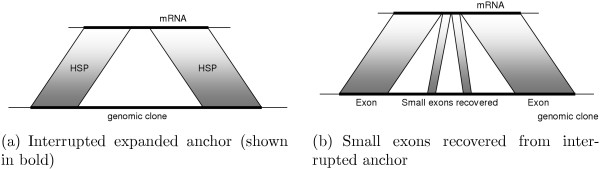
Recovery of small exons.

As an example of a successful anchor expansion, consider an HSP sequence interrupted by undetected short exon(s) (Figure 12). After combining interrupted unambiguous HSP sequences, as described [see Section *Implementation*], the small exons are recovered by processing the expanded anchors with our spliced alignment procedure.

**Figure 13:**
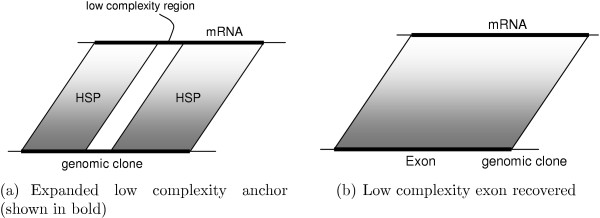
Handling of BLASTN HSP interrupted by low-complexity filtering.

Similarly, low-complexity regions may interrupt HSP sequencing in BLASTN results due to dust filtering. In this case, interrupted unambiguous HSP sequences are combined and the gap will be closed by sequence matching, resulting in a monolithic exon (Figure 13).

## Results

### Experiments with Genie learning set

GIGOgene was tested, along with Spidey, est2genome, Sim4, Galahad and BLAT on 462 mRNA transcripts of the human Genie multi-exon annotated learning set . We used transcripts corresponding to mRNA or CDS features in the Genie learning set annotation.

*Sensitivity *(*ESn*) and *specificity *(*ESp*) were calculated according to the formulas

ESn=TEAE     (3)
 MathType@MTEF@5@5@+=feaafiart1ev1aaatCvAUfKttLearuWrP9MDH5MBPbIqV92AaeXatLxBI9gBaebbnrfifHhDYfgasaacH8akY=wiFfYdH8Gipec8Eeeu0xXdbba9frFj0=OqFfea0dXdd9vqai=hGuQ8kuc9pgc9s8qqaq=dirpe0xb9q8qiLsFr0=vr0=vr0dc8meaabaqaciaacaGaaeqabaqabeGadaaakeaacqWGfbqrcqWGtbWucqWGUbGBcqGH9aqpdaWcaaqaaiabdsfaujabdweafbqaaiabdgeabjabdweafbaacaWLjaGaaCzcamaabmaabaGaeG4mamdacaGLOaGaayzkaaaaaa@398C@

ESp=TEPE     (4)
 MathType@MTEF@5@5@+=feaafiart1ev1aaatCvAUfKttLearuWrP9MDH5MBPbIqV92AaeXatLxBI9gBaebbnrfifHhDYfgasaacH8akY=wiFfYdH8Gipec8Eeeu0xXdbba9frFj0=OqFfea0dXdd9vqai=hGuQ8kuc9pgc9s8qqaq=dirpe0xb9q8qiLsFr0=vr0=vr0dc8meaabaqaciaacaGaaeqabaqabeGadaaakeaacqWGfbqrcqWGtbWucqWGWbaCcqGH9aqpdaWcaaqaaiabdsfaujabdweafbqaaiabdcfaqjabdweafbaacaWLjaGaaCzcamaabmaabaGaeGinaqdacaGLOaGaayzkaaaaaa@39B0@

Here *TE *is the number of accurately predicted exon boundaries, *AE *is the number of annotated exon boundaries in the Genie learning set, and *PE *is the number of predicted exon boundaries. Only internal exonic boundaries were considered. Results are summarized in Table 1.

**Table 1:** Comparative exon-level sensitivity and specificity for different programs on human Genie learning set

	*TE*	*AE*	*PE*	*ESn*	*ESp*
Galahad	4744	4909	4790	96.64%	99.04%
Spidey	4827	4909	4847	98.33%	99.59%
EST2genome	4742	4909	4752	96.60%	99.79%
Sim4	4837	4909	4845	98.53%	99.83%
BLAT	4832	4909	4902	98.43%	98.57%
GIGOgene	4864	4909	4865	99.08%	99.98%

This test is designed as evidence of general prediction quality of different gene structure annotation tools. GIGOgene has the highest sensitivity and specificity in this case, which highlights advantages of the approach we use.

### Experiments with micro-exon detection

We followed the Sim4 prediction compensating procedure described in [24] to identify human genes containing canonical micro-exons (3–12nt in our case). This way we were able to annotate 44 genes in the human DNA phase 3 database. Table 2 compares performance of different programs for a micro-exonic set of genes.

**Table 2:** Micro-exon gene set comparative level sensitivity and specificity for different programs

	*TE*	*AE*	*PE*	*ESn*	*ESp*
Galahad	1220	1422	1278	85.79%	95.46%
Spidey	1251	1422	1334	87.97%	93.78%
EST2genome	1270	1422	1318	89.31%	96.36%
Sim4	1278	1422	1326	89.87%	96.38%
BLAT	1375	1422	1424	96.69%	96.56%
GIGOgene	1420	1422	1422	99.86%	99.86%

This study shows that the GIGOgene program has the highest structural prediction sensitivity and specificity in this case. BLAT recovered 96.69% true exonic boundaries in the micro-exonic set, while other programs had fraction of true splice sites recovered no more than 90%, i.e. they most likely miss micro-exon(s) from their prediction.

### Experiments with non-canonical splice sites

According to [25] approximately 98.71% of all splice sites are reported to be canonical, 0.56% are in the biggest group of GC-AG non-canonical splices sites, and the remaining 0.76% consist of small groups of size no more than 0.05% each. Following the description in [25] we parsed the Human SpliceDB database of EST supported, corrected and GenBank High Throughput Genome sequencing projects (HTG) supported pairs of non-canonical splice sites. Then we aligned the pairs to the human RefSeq database using BLASTN to extract transcripts containing verified non-canonical splice sites. Found transcripts were BLAST-aligned to the NCBI human phase 3 DNA database to match corresponding gene-containing clones. We splice-aligned found transcripts and corresponding genomic clones using GIGOgene. A manual check on 108 gene structural predictions identified no problems on the GIGOgene side. A comparable performance study for other programs is shown in Table 3.

**Table 3:** Non-canonical gene set comparative level sensitivity and specificity

	*TE*	*AE*	*PE*	*ESn*	*ESp*
Galahad	2764	2896	2818	95.44%	98.08%
Spidey	2857	2896	2893	98.65%	98.76%
EST2genome	2788	2896	2888	96.27%	96.54%
Sim4	2868	2896	2893	99.03%	99.14%
BLAT	2880	2896	2987	99.45%	96.42%
GIGOgene	2896	2896	2896	100.00%	100.00%

In this study est2genome made a mistake in annotating virtually every non-canonical splice site while reinforcing canonical splice rule. Although BLAT was very sensitive in this experiment, it makes mistakes occasionally.

### Simulated EST experiment

In order to research the EST-related performance of different programs we introduced 4% noise in the Genie experiment discussed [see Subsection *Experiments with Genie learning set*]. Noise was equiprobably distributed between random nucleotide insertions, deletions and substitutions. Results of a simulated EST experiment are presented in Table 4.

**Table 4:** Noisy Genie experiment

	*TE*	*AE*	*PE*	*ESn*	*ESp*
Galahad	4531	4909	4655	92.30%	97.34%
Spidey	3547	4909	4759	72.26%	74.53%
EST2genome	4704	4909	4737	95.82%	99.30%
Sim4	4775	4909	4833	97.27%	98.80%
BLAT	3898	4909	17338	79.41%	22.48%
GIGOgene	4446	4909	4767	90.57%	93.27%

With simulated EST study our program performed worse than Sim4 and est2genome, about as well as Galahad, and substantially better than BLAT and Spidey, the programs that were specifically designed for mRNA/DNA spliced alignment. The reason for substantial quality loss with GIGOgene is in splice site annotation strategy. If we get a number of nucleotide inserts between exon boundaries in mRNA, they can be easily interpreted as micro-exon(s) with non-canonical splice sites, rather than reinforcing the GT-AG rule in a genomic clone as Sim4 and EST2genome do. That is why these two applications have rather poor performance in micro-exonic testing [see Subsection *Experiments with micro-exon detection*], where they sacrifice micro-exons to reinforce canonical splice rule.

### Run-time comparison

In Table 5 we compare running time for different programs required to annotate the set of micro-exon containing genes mentioned [see Subsection *Experiments with micro-exon detection*].

**Table 5:** Comparative time in seconds required by Pentium IV computer to annotate a set of genes containing micro-exons. BLASTN running time is included in GIGOgene timing.

Sim4	Spidey	BLAT	Galahad	GIGOgene	EST2genome
3.705 sec.	11.419 sec.	16.029 sec.	170.333 sec.	1504.444 sec.	5323.904 sec.

Run time comparison on the set of micro-exons indicates that our program runs faster than est2genome but slower than other tools we have looked at. By using splice-enhanced affine gap penalty global alignment we traded execution time for quality, compare to simpler heuristics used to predict splice sites in other tools.

### Chromosome 22 experiment

For this experiment we chose human chromosome 22 whole draft sequence NC_000022.8 from NCBI Genbank. A total of 506 transcripts were mapped to the chromosome by parsing human RefSeq flatfiles, but only 430 transcripts have corresponding genes annotated in NCBI Genbank.

We report running time for all 506 transcripts mapped to chromosome 22. For the GIGOgene program it took 12 hours 16 minutes 42 seconds to parse BLASTN results, while BLASTN took 9 days 18 hours 9 minutes 3 seconds to align transcripts to the chromosome (without dust filtering). Such a long running time could be explained by extensive low-complexity domains duplicated across the chromosome. BLASTN with low-complexity filtering took only 13 hours, 33 minutes and 18 seconds, but the following GIGOgene gene structural prediction was inferior to the results reported in Table 6. BLAT annotation took 12 hours 8 minutes 39 seconds (without dust filtering).

**Table 6:** Chromosome 22 prediction quality for 430 mapped transcripts with structural annotation

	*TE*	*AE*	*PE*	*ESn*	*ESp*
BLAT	7025	7088	8003	99.11%	87.78%
GIGOgene	7036	7088	7071	99.27%	99.51%

We report exon-level comparative performance of BLAT and GIGOgene in Table 6.

Results of BLAT and GIGOgene comparison on Chromosome 22 whole draft sequence annotation agree well with the previously observed tendency: with GIGOgene, gene structural prediction takes longer, compared to BLAT, and has higher prediction quality.

## Conclusion

Using a homology-based approach, we have designed a program for eukaryotic gene structural annotation. In case of mRNA/DNA spliced alignment we have been able to improve on exon-level sensitivity and specificity by addressing several possibilities of error. Program domain is limited to mRNA/DNA spliced alignment with a reasonable fraction of sequencing errors. Experiments on running time position our tool as a relatively slow utility for annotating specific cases of gene structural prediction.

Several published spliced alignment algorithms were mentioned [see Section *Background*]. Our splice-enhanced affine gap penalty global alignment in some ways similar to the spliced alignment of protein/DNA blocks described in the Procrustes paper [5]. The key differences in our implementation is that it works in linear memory and is effective in annotation of both canonical and non-canonical splice sites. Compared to protein-DNA alignment, it has finer granularity, which translates to smaller possibility for incorrect structural prediction, especially for micro-exons. We can also annotate both CDS and UTR regions, while protein-DNA homology programs, such as Procrustes [5] and Genomescan [26], are limited to CDS region only.

The stand-alone program version, web implementation interface, test results and manual for GIGOgene are available at .

## Availability and requirements

**Project name: **Good In Good Out gene structural prediction tool (GIGOgene)

**Project home page: **.

**Operating system: **Platform independent

**Programming language: **Java

**Other requirements: **Java 1.4.1 or higher

**License: **GNU Lesser General Public Licence

## Authors' contributions

AC and DQ conceptualized the project and set up computational facility. DQ implemented BLASTN SAX parser and made many valuable suggestions through the progress of our study. AC implemented and evaluated GIGOgene Java code. MP extensively edited the manuscript and made many important changes. HA helped to conceptualize the tool, provided general support and gave final approval of the version to be published. All authors read and approved the final manuscript.
